# Supplementary material for: Transcranial Direct Current Stimulation in Episodic Migraine: A Systematic Review and Meta-Analysis of Randomized Controlled Trials
Source: Med Sci (Basel). 2025 Jun 26;13(3):84. doi: 10.3390/medsci13030084 (PMC12286047; doi:10.3390/medsci13030084)
Supplement: Supplementary file 1 [file medsci-13-00084-s001.zip › medsci-3679606-supplementary.pdf]

Supplementary Table S1. Search strategy

**A. PubMed search until 07.07.2023**		
Number	Query	Number of results
#1	"Migraine Disorders"[Mesh]	31,770
#2	migrain*	47,104
#3	hemicran*	2,091
#4 (#1 OR #2 OR #3)	(("Migraine Disorders"[Mesh]) OR (migrain*)) OR (hemicran*)	48,596
#5	"Transcranial Direct Current Stimulation"[Mesh]	4,899
#6	transcranial direct current stimulation	8,020
#7	tDCS	8,729
#8 (#5 OR #6 OR #7)	(("Transcranial Direct Current Stimulation"[Mesh]) OR (transcranial direct current stimulation)) OR (tDCS)	8,729
#9 (#4 AND #8)	((("Migraine Disorders"[Mesh]) OR (migrain*)) OR (hemicran*)) AND ((("Transcranial Direct Current Stimulation"[Mesh]) OR (transcranial direct current stimulation)) OR (tDCS))	86
**B. Embase search via Ovid until 07.07.2023**		
#1	migraine.mp. or migraine/	90,090
#2	migrain$.mp.	91,046
#3	hemicrania.mp.	1,768
#4	#1 OR #2 OR #3	91,589
#5	transcranial direct current stimulation.mp. or transcranial direct current stimulation/	11,915
#6	tDCS.mp.	10,038
#7	#5 OR #6	12,998
#8	#4 AND #7	228
